# Understanding out-of-pocket spending and financial hardship among patients who succumb to cancer and their caregivers

**DOI:** 10.1186/s13584-021-00511-8

**Published:** 2022-01-03

**Authors:** Aviad Tur-Sinai, Damien Urban, Daniel Azoulay, Gil Bar-Sela, Netta Bentur

**Affiliations:** 1 Department of Health Systems Management, The Max Stern Yezreel Valley College, 1930600 Yezreel Valley, Israel; 2 School of Nursing, University of Rochester Medical Center, Rochester, NY USA; 3 Department of Oncology, Sheba Medical Center, Tel Hashomer, Israel; 4 Unit for Palliative Care, Hadassah Medical Center, Mount Scopus, Jerusalem, Israel; 5 Oncology and Hematology Division, Emek Medical Center, Afula, Israel; 6 The Stanley Steyer School of Health Professions, Sackler Faculty of Medicine, Tel-Aviv University, Tel-Aviv, Israel

**Keywords:** Out-of-pocket spending, Cancer, Financial burden, Private caregiver, Medicines

## Abstract

**Background:**

In most countries, including those with national health insurance or comprehensive public insurance, some expenses for cancer treatment are borne by the ill and their families.

**Objectives:**

This study aims to identify the areas of out-of-pocket (OOP) spending in the last half-year of the lives of cancer patients and examine the extent of that spending; to examine the probability of OOP spending according to patients’ characteristics; and to examine the financial burden on patients’ families.

**Methods:**

491 first-degree relatives of cancer patients (average age: 70) who died 3–6 months before the study were interviewed by telephone. They were asked about their OOP payments during the last-half year of the patient's life, the nature of each payment, and whether it had imposed a financial burden on them. A logistic regression and ordered logit models were used to estimate the probability of OOP expenditure and the probability of financial burden, respectively.

**Results:**

Some 84% of cancer patients and their relatives incurred OOP expenses during the last half-year of the patient’s life. The average levels of expenditure were US$5800on medicines, $8000 on private caregivers, and $2800 on private nurses. The probability of paying OOP for medication was significantly higher among patients who were unable to remain alone at home and those who were less able to make ends meet. The probability of spending OOP on a private caregiver or private nurse was significantly higher among those who were incapacitated, unable to remain alone, had neither medical nor nursing-care insurance, and were older. The probability of a financial burden due to OOP was higher among those unable to remain alone, the incapacitated, and those without insurance, and lower among those with above-average income, those with better education, and patients who died at home.

**Conclusions:**

The study yields three main insights. First, it is crucial that oncology services provide cancer patients with detailed information about their entitlements and refer them to the National Insurance Institute so that they can exercise those rights. Second, oncologists should relate to the financial burden associated with OOP care at end of life. Finally, it is important to sustain the annual increase in budgeting for technologies and pharmaceuticals in Israel and to allocate a significant proportion of those funds to the addition new cancer treatments to the benefits package; this can alleviate the financial burden on patients who need such treatments and their families.

## Introduction

Cancer is one of the costliest illnesses that a person can encounter [29] and the costs of its treatment are rising more briskly than in many other areas of healthcare [28]. Although cancer-related healthcare costs vary widely among countries [5, 11, 18, 25], including countries that have universal healthcare systems, statutory health insurance, and/or strong health-technology assessment processes, supplemental out‐of‐pocket (OOP) expenses for cancer patients are common [26]. Health-insurance systems and insurers are increasingly passing costs of care onto patients by raising deductibles, introducing copayments, and taking out coinsurance [13]. This creates significant discrepancies in the cost of cancer medication to patients because even if a given pharmaceutical comes at a fixed price, it varies relative to household income and the expense may affect persons with cancer in different ways (Davidoff et al. 2013) [1]. Therefore, cancer care foists a substantial financial burden not only on society and healthcare systems but also on patients, their families, and their relatives [2, 30].

In the past decade, many researchers around the world have described the physical, mental, and financial struggles of patients and their families in coping with the need to pay for expensive medicines and services in an attempt to save or prolong their dear ones’ lives [19]. In the United States, these expenses add up to 20–30% of annual household income and impose a financial burden on one-third to two-thirds of cancer patients and their families [6, 8, 10, 15, 24, 27, 33, 34]. In a study from the United States among people with stomach cancer, 38% of respondents reported a financial burden that caused them to amass debts, sell a dwelling, or take loans from family members and friends [23]. In other papers, 31% reported that the diagnosis and treatment of cancer imposed a financial burden on them [10]. Ubel et al. [27], basing themselves on data from the National Center for Health Statistics for 2009, reported that 30% of holders of private insurance and 38% of those with public insurance aged 65 or less were financially burdened by some form of expenditure on healthcare. Apart from the financial burden, OOP spending may cause patients and their families mental distress, anxiety, depression, impairment of quality of life, and long-term financial disadvantage [31].

OOP spending by cancer patients and their families falls into direct and indirect categories [32]. Direct expenditure relates mainly to medicines not covered by National Health Insurance or other insurance plans, and for at-home nursing care when the patient has difficulty in functioning due to weakness. Indirect spending includes paying for transport to the hospital, sometimes by taxi or ambulance, food and board for family members if care is given far from their place of residence; and other outlays. Some studies also include lost work days of patients and their families [7, 32].

OOP expenses associated with cancer treatment also raise difficult ethical questions relating to the need to make decisions that will affect the continuation of the families’ lives, such as whether to sell a house or take a loan to finance care. In the literature, this is called “financial toxicity” because, like the physical toxicity that cancer treatment may cause, some people also struggle to cope with the suffering associated with the financial shortfall and psychological stress occasioned by the steep expenses attached to oncological care [34].

To the best of our knowledge, there are no data on OOP spending by people with cancer and their families in Israel. Much has been published about cancer patients’ financial rights, such as an exemption from and a ceiling on copays for medicines, medical services, pharmacological food, dental care, and other services, but research has not addressed itself to the extent of their spending and that of their families, and their financial burden. A series of articles in *Ha’aretz* by the journalist Ronny Linder-Ganz [17]*,* titled “Your Life for a Million Shekels: Cancer Medications That Can Save My Father,” shed light on the financial burden, the mental distress, and the anguish that many people in Israel experience.

Many studies have repeatedly illuminated the dire financial consequences of the cost of cancer medications for patients and their families [4]. Most of them, however, investigated cancer survivors and, to the best of our knowledge, overlooked the financial burden on family members until the death of their loved ones, even though this burden may persist and even continue to expand. This demonstrates the immense importance of taking into account not only patients’ OOP outlays and their financial consequences but also the ways in which their entire families cope and live with the outcome.

Accordingly, the current study has three main goals: to identify the areas of OOP expenditure on the care of people who died from cancer in the last half-year of their lives and determine the extent of spending for medicines and nursing care; to determine the likelihood of OOP spending parsed by patients’ characteristics; and to examine the financial burden of caregiving on the family.

## Methods

This was a retrospective cross-sectional study. Its target population comprised persons aged 23 and over, Jews and Arabs, who had died from cancer three to six months before the study and had been treated at Sheba Medical Center in Tel Hashomer, Emek Medical Center in Afula, Hadassah Medical Center, or Shaare Zedek Medical Center in Jerusalem during 2018–2019. The names of consecutively 1000 patients who had died about half a year before the survey at these departments were retrieved from the centers’ medical records, as were the details of their primary first-degree relatives.

The actual study population was composed of patients’ primary first-degree relatives whose names appeared in the medical records. They, and not the patients themselves, were chosen for three reasons. First, it is too burdensome for people in terminal condition to be interviewed because many are too weak to speak and/or not adequately alert; some are no longer aware of the financial and administrative aspects of their care. Second, to capture all OOP expenses, the interview should take place only after the end of the patient's life. Third, to examine the financial burden of caregiving, it is the relatives of persons who had passed away, who had assumed most of the burden of caregiving, who should be interviewed.

A medical secretary contacted the primary first-degree relatives by telephone, briefly explained them the aim of the survey, and asked them for their consent to be interviewed by telephone. Of those contacted, 491 family caregivers answered in the affirmative (49% of the study target population). More than half (55%) of those who refused to be interviewed said that it is too difficult for them to speak about the last period of their loved one’s life, 29.5% said that they had hardly had OOP expenses because their loved ones’ condition had deteriorated very quickly, and 15.5% said that they did not remember their expenses.

The primary first-degree relatives were interviewed by skilled interviewers who were directed by the investigators. Before the interview began, the interviewers read to them the consent document and they reconfirmed their consent to be interviewed. They were interviewed via a closed-ended structured questionnaire that included items about their OOP payments and the financial burden they had incurred on account of medicines, caregivers, private nurses, and other expenses for which they had received no reimbursement from any source in the last half-year of the patients’ lives. The financial burden was measured by means of one question that was developed for the current study: To what extent did OOP expenditure create a financial burden for the patient and the whole family? The answers ranged from 1, not at all, to 4, a heavy burden.

The two dependent variables in this study were (1) the probability of spending out of pocket on medications, private caregiver, and private nurse; and (2) the extent to which these three kinds of spending impose a general burden on the patient’s family. These variables were examined by means of three questions. The relatives were asked whether they had incurred any expenses for each of the items. Those who answered in affirmative for either item (e.g., medication) were asked how much they had spent on it. The amount of OOP spending for each item in ILS (Israeli currency) was converted to USD on the basis of the official exchange rate published by the Bank of Israel on August 1, 2021. Then the relatives were asked about the extent to which this spending had been burdensome to them.

The independent variables included in the investigation were patient’s gender (a dichotomous variable: 0 = male; 1 = female), patient’s age (a continuous variable), patient’s education (1 = 1–4 year, 2 = 5–8 years, 3 = 9–12 years, 4 = 13 + years), patient’s difficulties in functioning (patient’s incapacity) (1 = able to perform all Activities of Daily Living (ADL); 2 = has difficulty in performing Activities of Daily Living alone; 3 = totally unable to perform Activities of Daily Living); patient’s unable to remain alone during the day (a dichotomous variable: 0 = able to remain alone during the day; 1 = unable to remain alone during the day); patient’s health-insurance coverage (0 = private health insurance or nursing-care insurance;1 = no private health insurance or nursing-care insurance;); patient’s household’s economic capacity (household’s ability to make ends meet: 1 = with great difficulty, 2 = with some difficulty, 3 = pretty easily, 4 = easily); patient’s household income (1 = far below average, 2 = somewhat below average, 3 = around the average, 4 = somewhat above average, 5 = far above average); dying at home (a dichotomous variable: 0 = in hospital/nursing facility/inpatient hospice; 1 = at patient’s own home or in other person’s home).

The data were analyzed by means of the STATA 15 program at two-tailed significance (*p* < 0.05). Descriptive statistical indicators were used first, followed by a bivariate analysis. Then, to test the probability of OOP expenditure and the relation between it and characteristics of the population and the financial burden, logistic and ordered logit regressions were conducted.

The study was conducted and was approved by the four centers’ Helsinki committees (4889–18-SMC, 0022–19-EMC, 0201–19-HMO, 0285–18-SZMC).

## Results

The deceased patients’ average age was seventy (S.D. = 13.14), 52% were women, and 49% had academic education. Some 42% were totally or almost totally incapacitated, 32% functioned with difficulty, and one-fourth were able to carry out Activities of Daily Living. Around 56% of patients’ households had strong or rather strong economic capacity; all the others reported some or considerable difficulty in this regard. Eighty-four percent of those who died from cancer incurred OOP expenses on care during the patients’ last half-year of life (Table 1).

**Table 1:** Demographic, socioeconomic, and functional characteristics of persons who died from cancer (percent), N = 491

	Had no OOP spending	Had OOP spending	F/χ^2^	Total
	N = 79 16.09%	N = 412 83.91%		N = 491 100%
Age (mean and S.D.)	66.2 (13.9)	70.3 (12.9)	1.25	69.5 (13.14)
Gender			1.431	
Male	18.14	81.86		48.3
Female	14.17	85.83		51.7
Education			1.775	
1–4 years	5.19	2.77		3.16
5–8 years	9.09	7.81		8.02
9–12 years	41.56	39.80		40.08
13 + years	44.16	49.62		48.73
Functional condition			30.213***	
Totally or almost totally incapacitated	10.19	89.81		41.96
Difficulties in doing ADL alone	11.39	88.61		32.18
Able to do ADL	31.50	68.50		25.87
Able to remain at home alone	33.72	66.28	13.546***	17.52
Unable	12.35	87.65		82.48
Economic capacity** (**household’s ability to make ends meet)			1.346	
With great difficulty	8.11	11.73		11.14
With some difficulty	29.73	32.53		32.07
Pretty easily	29.73	27.20		27.62
Easily	32.43	28.53		29.18
Insurance			2.001	
Has supplemental health-insurance coverage	14.37	85.63		65.17
No insurance	19.30	80.70		34.83

*p < 0.05, **p < 0.01, ***p < 0.001

**Fig. 1:**
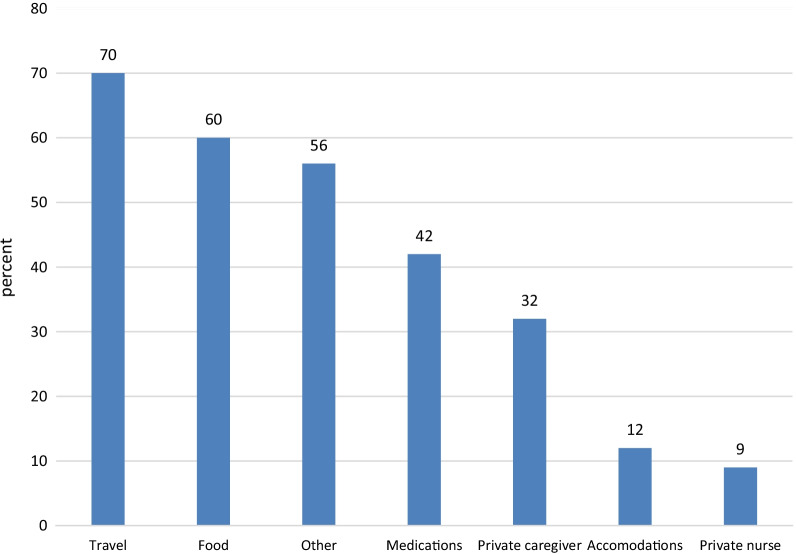
Out-of-pocket spending by persons who died from cancer and by their families in the patients’ last half-year of life (percent), N = 491. “Other” denotes financial expenses for other needs occasioned by the illness (e.g., consulting with a psychologist and mental care)

As for direct caregiving expenses, 42% paid OOP for medicines, 32% for a private caregiver, and 9% for a private nurse. Families also ran up indirect expenses for care, mainly when the patient was in hospital. Thus, 70% paid OOP for traveling to the hospital and back, 60% for food away from home, and 12% for overnight accommodations (Fig. 1).

The probability of having to pay out of pocket for a medication was high and significant among patients who were unable to remain alone at home (OR = 1.268, 95% CI = 1.031–2.129, *p* < 0.01) and those less able to make ends meet (OR = 0.798, 95% CI = 0.643–0.990, *p* < 0.05). The likelihood of spending OOP for a private caregiver was high and significant among the incapacitated (OR = 1.371, 95% CI = 0.948–1.985, *p* < 0.1), among patients who could not remain at home alone during the day in the last half-year of their lives (OR = 4.305, 95% CI = 2.012–9.212, *p* < 0.01), and for patients who had neither medical nor nursing-care insurance (odds ratio = 1.893, 95% CI = 1.069–3.349, *p* < 0.05). The likelihood of OOP expenditure on a private nurse was significant and rose commensurate with the patient’s age (OR = 1.028, 95% CI = 0.999–1.059, *p* < 0.1) and for patients who lacked private insurance (medical or nursing-care) (OR = 3.299, 95% CI = 1.312–8.291, *p* < 0.05) (Table 2).

**Table 2:** Probability of out-of-pocket expenditure, by purposes (odds ratios) (95% CI) (Logit model)

	Medications	Private caregiver	Private nurse
Female (0 = male; 1 = female)	1.179 (0.776–1.790)	2.274*** (1.350–3.830)	1.331 (0.662–2.675)
Patient’s age	0.991 (0.976–1.007)	1.066*** (1.041–1.091)	1.028* (0.999–1.059)
Patient’s incapacity (1: able to perform all Activities of Daily Living (ADL) – 3: totally unable to perform ADL)	1.010 (0.750–1.360)	1.371* (0.948–1.985)	1.234 (0.742–2.050)
Unable to remain alone during the day (0 = able to remain alone during the day; 1 = unable to remain alone during the day)	1.268*** (1.031–2.129)	4.305*** (2.012–9.212)	1.373 (0.542–3.474)
Has supplemental health-insurance coverage (0 = yes; 1 = no)	0.885 (0.569–1.377)	1.893** (1.069–3.349)	3.299** (1.312–8.291)
Household’s economic capacity (1: with great difficulty – 4: easily)	0.798** (0.643–0.990) (0.09)	1.029 (0.794–1.333) (0.14)	0.854 (0.598–1.219) (0.16)
Pseudo R2	0.1702	0.1782	0.1558
N	379	379	379

*p < 0.10, **p < 0.05, ***p < 0.01

In regard to the economic burden borne by those who incurred OOP expenses, one-third of those who spent OOP on a private caregiver did not find this expense burdensome, roughly 40% of those who spent OOP on a private nurse did not see this as burdensome, while among those who spent OOP on medicines, one in five felt no financial burden at all on this account. More than 55% of those who spent OOP on a private caregiver considered it very burdensome, roughly 41% of those who spent OOP on a private nurse considered it very burdensome, whereas more than half of those who spent OOP on medicines found it severely burdensome (Table 3).

**Table 3:** Prevalence of persons who spent out of pocket on health necessities, by extent of financial burden (percent)

	No burden	Minor burden	Heavy burden	χ^2^
Private caregiver	30.20	14.09	55.70	298.00***
Private nurse	38.64	20.45	40.91	
Medications	19.05	28.57	52.38	

The average outlay during the six-month period for medicines was US$5800 (S.D. $8500; median $1800). The average expenditure on a private caregiver during the last half-year of the patients’ lives was $8000 (S.D. $7300; median $6000) and on a private nurse $2800 (S.D. $5000; median $440). The average expenditure on medicines, a private caregiver, or a private nurse during the six-month period among those who reported this expenditure as not financially burdensome at all was USD 2449, USD 6277 and USD 591, respectively. The average expenditure on medicines, a private caregiver, or a private nurse during the six-month period among those who reported this expenditure highly burdensome was USD 11,144, USD 8941 and USD 5153, respectively (Table 4).

**Table 4:** Out-of-pocket expenditure on necessities matters, by extent of financial burden (USD)

	No burden	Minor burden	Heavy burden
Private caregiver	6277.964 (± 6509.04) [5006.068]	6367.315 (± 8926.5) [7584.951]	8941.93 (± 7410.62) [6674.757]
Private nurse	591.6262 (± 388.143) [197.2087]	907.4363 (± 2227.54) [667.4757]	5153.101 (± 6513.61) [1516.990]
Medications	2449.724 (± 3925.86) [834.3447]	7205.704 (± 10,542.4) [1820.388	11,144.21 (± 8470.69) [3033.981

The numbers in parentheses (the first row) represent the standard deviation. The numbers in square brackets (second row) present the median

The probability of a financial burden on patients and relatives due to OOP spending on a private caregiver was higher among patients who were unable to remain alone during the day (OR = 3.086, 95% CI = 1.126–8.453, *p* < 0.05). The inclusion of information about patients’ having health insurance shows that the financial burden was greater among those who had no insurance (OR = 2.112, 95% CI = 0.879–5.074, *p* < 0.1) than among those who carried supplemental health coverage. It is the inclusion of information about the household’s income that makes the insurance coverage not significant, probably due to the high correlation between them. It shows that the more easily a household makes ends meet or if it has above-average income, the less likely it is that the patient and their family will incur a financial burden for OOP spending on a caregiver (OR = 0.646, 95% CI = 0.448–0.930, *p* < 0.05 and 0.669, 95% CI = 0.484–0.925, *p* < 0.05, respectively). In addition to all these, it is found that the probability of a financial burden is smaller when the patient dies at home (OR = 0.512, 95% CI = 0.244–1.072, *p* < 0.1 in Model 1; OR = 0.558, 95% CI = 0.278–1.083, *p* < 0.1 in Model 2) (Table 5).

**Table 5:** Probability of financial burden on patient and family members due to out-of-pocket expenditure on private caregiver (odds ratio) (95% CI) (Ordered Logit model)

	Model 1	Model 2
Unable to remain alone during the day (0 = able to remain alone during the day; 1 = unable to remain alone during the day)	3.086** (1.126–8.453)	2.146 (0.829–5.550)
Dying at home (0 = in hospital/nursing facility/inpatient hospice; 1 = at patient’s own home or in other person’s home)	0.512* (0.244–1.072)	0.558* (0.278–1.083)
Has supplemental health-insurance coverage (0 = yes; 1 = no)	2.112* (0.879–5.074)	1.970 (0.845–4.594)
Household’s economic capacity-(1: with great difficulty–4: easily)	0.646** (0.448–0.930)	
Household income above average (1: far below average–5: far above average)		0.669** (0.484–0.925)
Pseudo R2	0.1633	0.1576
N	133	138

*p < 0.10, **p < 0.05, ***p < 0.01

The probability of a financial burden on a patient and his or her family due to OOP payment for medicines is smaller among well-educated patients (OR = 0.0.543, 95% CI = 0.299–0.985, *p* < 0.05 in Model 1; OR = 0.560, 95% CI = 0.313–0.998, *p* < 0.05 in Model 2) and higher among the incapacitated (OR = 1.377, 95% CI = 0.960–1.975, *p* < 0.1) (Table 6). Furthermore, the inclusion of information about the household’s financial situation shows that the more easily the household makes ends meet or has above-average income, the less likely it is to incur a financial burden by paying OOP for a caregiver (OR = 0.527, 95% CI = 0.387–0.720, *p* < 0.01 in Model 1 and OR = 0.679, 95% CI = 0.518–0.892, *p* < 0.01 in Model 2, respectively) (Table 6).

**Table 6:** Probability of financial burden on patient and family members due to out-of-pocket expenditure on medications (odds ratio) (95% CI) (Ordered Logit model)

	Model 1	Model 2
Patient’s education (1: 1–4 year–4: 13 + years)	0.543** (0.299–0.985)	0.560** (0.313–0.998)
Patient’s incapacity (0: able to perform all Activities of Daily Living (ADL)–2: totally unable to perform ADL)	1.377* (0.960–1.975)	1.265 (0.882–1.814)
Household’s economic capacity (1: with great difficulty–4: easily)	0.527*** (0.387–0.720)	
Household income above average (1: far below average–5: far above average)		0.679*** (0.518–0.892)
Pseudo R2	0.1852	0.1501
N	188	188

*p < 0.10, **p < 0.05, ***p < 0.01

The probability of a financial burden on a patient and his or her family due to any of these three OOP payments (medicines, private caregiver, or private nurse) is higher among the incapacitated (OR = 2.611, 95% CI = 1.517–4.495, *p* < 0.01 in Model 1, OR = 1.857, 95% CI = 1.090–3.164, *p* < 0.01 in Model 2). Furthermore, the more easily a household makes ends meet or if it has above-average income, the less likely it is to incur a financial burden by paying OOP for any of these health necessities (OR = 0.553, 95% CI = 0.422–0.724, *p* < 0.01 and OR = 0.688, 95% CI = 0.546–0.868, *p* < 0.01, respectively) (Table 7).

**Table 7:** Probability of financial burden on patient and family members due to out-of-pocket expenditure on medications, private caregiver, or private nurse (odds ratio) (95% CI) (Ordered Logit model)

	Model 1	Model 2
Patient’s education (1: 1–4 year–4: 13 + years)	0.728 (0.437–1.213)	0.767 (0.467–1.259)
Unable to remain alone during the day (0 = able to remain alone during the day; 1 = unable to remain alone during the day)	2.611*** (1.517–4.495)	1.857*** (1.090–3.164)
Has supplemental health insurance coverage (0 = yes; 1 = no)	0.725 (0.426–1.232)	0.827 (0.495–1.381)
Household’s economic capacity (1: with great difficulty – 4: easily)	0.553*** (0.422–0.724)	
Household income above average (1: far below average–5: far above average)		0.688*** (0.546–0.868)
Pseudo R2	0.1704	0.1395
N	263	267

*p < 0.10, **p < 0.05, ***p < 0.01

## Discussion

The study shows that people who died from cancer and their families spent out of pocket for reasons related to the illness and its treatment during the patient’s last half-year of life. Forty-two percent of the respondents spent OOP on medicines, 32% spent on a private caregiver, 9% spent on a private nurse and a majority had other unreimbursed expenditure on matters such as travel (70%), food (60%) and room and board away from home. Among those who refused to take part in the study, 29.5% reported negligible OOP expenses because their loved ones’ condition had deteriorated very quickly.

The two main determinants of OOP expenditure at the end of life of persons who succumbed to cancer and of their families were medications and private caregivers. The finding that a large share of people in Israel spent out of pocket for medications is consistent with findings from Europe and the United States that trace the main OOP expenditure for people with cancer to medications not included in public coverage, whereas spending for nursing is smaller in most countries 26. It was found in this study, however, that the average outlay for a private caregiver is the highest among the expenditure items even though the standard deviation of OOP spending, especially on medications, is very wide, possibly attesting to large differences in expenditure. One explanation for this may have to do with the total separation of entitlement to medical care and entitlement to National Insurance funding of a caregiver in Israel. Namely, the entitlement to medical care in Israel is universal whereas eligibility for a caregiver is based on meeting criteria. In Europe and, to some extent, in the United States (chiefly in Medicare), in contrast, the same authorities fund and deliver most medical and nursing services for the ill [11]. In this context, it is important to note the need for caution and the difficulty of comparing findings from different countries because the differences in spending on various services may be related to the pricing of treatments and medicines in different places [5, 28].

The findings reported above specify advanced age, incapacity, and inability to remain at home alone as the main determinants of OOP expenditure [15, 23]. The findings of studies performed elsewhere, identify financial hardship and lack of health insurance as the main determinants of the likelihood of OOP spending for the treatment of cancer [12, 35]. The difference may trace to the gap in patients’ functioning ability due to the need for relatives’ assistance during their illness.

From a policy perspective, the current study assesses the subjective evaluation of "burden" rather than measuring the percentage of income consumed by these OOP expenditures. In this study, more than half of family members described OOP expenditure as financially burdensome to them. This indicates the existence of the “financial toxicity” phenomenon in Israel, too. These findings resemble those from elsewhere about the financial burden that the high cost of care inflicts [6, 8, 10, 15, 24, 27, 33, 34].

More than a decade ago, in view of the negative financial consequences of pharmacological care for people with cancer and their families, the Cost of Care Task Force at the American Society of Clinical Oncology (ASCO) singled out a patient-physician conversation about costs as a critical feature of high-quality care [20]. Ten years later, the President’s Cancer Panel [21] acknowledged the importance of addressing the high prices of cancer medications as a matter of national priority. ASCO, too, related to steep OOP expenditure in a position statement that it released about the affordability of cancer drugs [3]. The findings of the current study—that a large proportion of people in Israel incur significant OOP expenditure on cancer medications and that this expenditure foists a perceptible financial burden on them—reinforce these arguments. Another possible corollary of these findings is the importance of enhancing awareness, both among medical staff and among patients and their relatives, of the advantages of palliative care in the early stages of incurable illnesses.

Researchers in other countries also found a connection between financial burden and psychological distress, anxiety and depression, impairment of quality of life, and the debts and destitution that family members face after the patient’s death [31]—phenomena that were not examined in the current study. Future research may shed additional light on this important matter, examining the implications of the financial outlay for the care of the ill family member for relatives and main caregivers after their dear ones’ death and possibly indicating how well they manage to recover financially after the terminal event.

The current study has several noteworthy limitations. First, it may be argued that family members do not know enough about patients’ OOP expenses. Israel, however, is typified by close relations of family support, responsibility, and involvement [16]. In Israel, one presumes, first-degree relatives who accompany patients in their final months know about the expenses and are involved in covering them. Indeed, a study published in Israel shows that patients’ family members describe themselves, in the plural, as having been cancer patients [22]. The possibility always exists, however, that family members do not remember all details of all expenditure. Second, since it is forbidden to give any information about patients (and their family members) before obtaining consent, we could not compare the characteristics of those who refused to be interviewed with those of the study population. As a result, a possible selection bias may be considered. Third, reporting on financial burden may be subjective; indeed, two households with similar income may report on their burden experience differently. Since we did not asked about the respondents’ confidence in the accuracy of the information, a recall bias may be considered. Fourth, having been unable to stratify our analyses by types of cancer, we could not provide more specific policy implications. Fifth, the use of a four-point scale to measure the extent of the financial burden (a core variable in this study) may not extract variability among subjects.

## Conclusions and recommendation

This study yields several insights. First, since many patients and their families incur non-negligible out of pocket outlays on medicines and services not covered by National Health Insurance, their financial burden may be highly onerous. This burden could be seriously disadvantageous especially to poorer persons but could also be significant for others who lack private insurance and who need a paid caregiver. The problem stems partly from the fragmentation of healthcare and social authorities in Israel: healthcare services are delivered by the HMOs whereas nursing care is arranged through the National Insurance Institute. Since all interaction and communication with cancer patients during their illness takes place vis-à-vis the medical services, many patients and families are unaware of their eligibility for National Insurance benefits. Such is the case even though the National Insurance Institute has established a “green lane” for persons with cancer in order to help them exercise their eligibility for benefits and nursing-care services. Therefore, the oncology services should expand their roles in order to take a holistic view of care, including its related financial burden. It is important to step up the healthcare services’ involvement in giving information about patients’ eligibility for a caregiver, referring them to the National Insurance Institute, and helping them to exercise their rights. This role—making sure that care is given within a holistic view of the patient and their family—should be one of the main duties of the social worker on the oncology team.

Second, the prevalence of OOP spending on medicines in the last stages of patients’ lives strongly underscores the need to address and discuss financial considerations along with those relating to quality of care. Oncologists should play a central role not only in delivering high-quality medical treatment but also in coordinating different aspects of patient care such as helping to contain the financial burden. Although the role of oncologists in this discussion is clear and gradually being accepted, their involvement in explaining OOP expenditures on medicines still leaves room for improvement. Explaining the complex interactions of cost and clinically meaningful outcomes is no easy task; therefore, oncologists need education in developing skills that would enable them to communicate costs more openly and consider the cost of a treatment when prescribing it. This approach has been endorsed by the American Society of Clinical Oncology, which recommends the development of a guideline statement on cancer costs [14] and defines financial counseling as an integral part of cancer care [24]. Moreover, ASCO suggests [9] that oncologists should be taught not only how to discuss cost affordability but also how to discuss survival life expectancy when recommending out-of-pocket medicines. More research is needed in Israel to elucidate the influence of oncologists, the challenges they face in communicating about out-of-pocket costs of care, and what can be done to help them overcome their avoidance of the topic. Future research should also investigate whether OOP spending and lack of communication with oncologists not only create a burden but also cause cancer patients to forgo medications that the basket of insured services does not cover.

Finally, it is important to sustain the tendency to allot a meaningful portion of the annual increase in the budgeting of technologies and pharmaceuticals in Israel to the inclusion of new medicines for the treatment of cancer in order to alleviate the financial burden on those who need them.

## Data Availability

The dataset is available from the corresponding author upon request.

## Abbreviation

OOP: Out of pocket
